# Effects of Artificial Ligaments with Different Porous Structures on the Migration of BMSCs

**DOI:** 10.1155/2015/702381

**Published:** 2015-05-28

**Authors:** Chun-Hui Wang, Wei Hou, Ming Yan, Zhong-shang Guo, Qi Wu, Long Bi, Yi-Sheng Han

**Affiliations:** Department of Orthopaedics, Xijing Hospital, Fourth Military Medical University, No. 15 West Changle Road, Xi'an 710032, China

## Abstract

Polyethylene terephthalate- (PET-) based artificial ligaments (PET-ALs) are commonly used in anterior cruciate ligament (ACL) reconstruction surgery. The effects of different porous structures on the migration of bone marrow mesenchymal stem cells (BMSCs) on artificial ligaments and the underlying mechanisms are unclear. In this study, a cell migration model was utilized to observe the migration of BMSCs on PET-ALs with different porous structures. A rabbit extra-articular graft-to-bone healing model was applied to investigate the *in vivo* effects of four types of PET-ALs, and a mechanical test and histological observation were performed at 4 weeks and 12 weeks. The BMSC migration area of the 5A group was significantly larger than that of the other three groups. The migration of BMSCs in the 5A group was abolished by blocking the RhoA/ROCK signaling pathway with Y27632. The *in vivo* study demonstrated that implantation of 5A significantly improved osseointegration. Our study explicitly demonstrates that the migration ability of BMSCs can be regulated by varying the porous structures of the artificial ligaments and suggests that this regulation is related to the RhoA/ROCK signaling pathway. Artificial ligaments prepared using a proper knitting method and line density may exhibit improved biocompatibility and clinical performance.

## 1. Introduction

Anterior cruciate ligament (ACL) rupture is the most common ligament disorder in daily life [1]. The ACL is an intra-articular ligament that connects the femur to the tibia to maintain the stability of the knee joint [2]. Because of the poor self-healing ability of an injured ACL, surgical ACL reconstruction is usually required [3]. In the USA, approximately 6‰ of the population suffers pain due to ACL injury each year, half of whom require surgical ACL reconstruction [4–7]. Autologous and allogeneic grafts are widely utilized in traditional ACL replacement and reconstruction methods, but they are limited by donor-site morbidity and a long rehabilitation period [8]. Since the 1980s, artificial ligaments, such as Gore-Tex and Dacron ligament prostheses, have increased in popularity. Deterioration is a typical long-term clinical outcome due to mechanical mismatch and limited graft-to-bone healing [9]. In 1992, ligament advanced reinforcement system (LARS) ligaments composed of polyethylene terephthalate (PET) were developed [10, 11]. LARS ligaments are approved for marketing by the United States Food and Drug Administration (FDA) and have been applied clinically for decades [12, 13] but have some disadvantages [14]. Guidoin et al. observed that scar tissue, macrophages, and giant cells could infiltrate between the fibers, resulting in ligament failure [15]. The LARS ligament is not biodegradable, complicating tissue ingrowth to form a new nature ligament [16]. Although inducing tissue ingrowth is very important for the biocompatibility of implants [17], tissue ingrowth is often random and is affected by the surface topography, pore diameter, and porosity of the materials [18, 19]. For scaffolds of different porosities, the cellular responses to the implant differ [20]. After the implantation of artificial ligaments, bone marrow mesenchymal stem cells (BMSCs) are the primary cell type in the implanted area. Implants with appropriate surface topography, pore diameter, and porosity may guide the migration of BMSCs and encourage bone tissue ingrowth and promote graft-to-bone healing.

In general, cellular migration is a coordinated cyclic process of polarization, protrusion, attachment, contraction, and rear release [21, 22]. The Rho family of small guanosine triphosphatases (including RhoA, Rac, and Cdc42) is involved in one of the most important molecular signaling pathways mediating cellular migration [23–25]. Cdc42 is required for the cell polarization and filopodia formation that influence protrusions. Rac is essential for lamellipodia formation and forward movement, while RhoA regulates actin stress fibers and focal adhesion formation [26, 27]. Rac and Cdc42 can active RhoA to regulate trailing edges formation at the back of the cell [28, 29]. These three proteins cooperate with each other for efficient cellular migration. Rho-associated kinase (ROCK), a downstream effector of Rho that is activated by RhoA, phosphorylates the downstream substrate myosin light chain phosphatase (MLCP) directly or indirectly [27, 30, 31]. ROCK can also decrease cofilin activity to increase random migration [32]. Finally, RhoA and ROCK signaling regulate tail retraction and integrin adhesion at the rear of cells [33]. Y27632, a specific inhibitor of ROCKs, inhibits the RhoA kinase family and influences stress fibers, vinculin and myosin light chain phosphatase [34].

The purpose of this study was to investigate cellular migration ability on PET-based artificial ligaments (PET-ALs) with different porous structures prepared using different knitting methods. We hypothesized that cellular migration is regulated by the porosity of artificial ligaments and that differences in BMSC migration are attributable to the RhoA/ROCK signaling pathway. Thus, selection of a proper knitting method and line density for artificial ligaments may enable improved biocompatibility and clinical performance.

## 2. Materials and Methods

### 2.1. Preparation of Grafts and Mode

To obtain PET-ALs with different porous structures, we employed 500 PET fibers (90% crystalline, diameter: 28.46 ± 1.04 *μ*m) in a bundle with a line density of 5000 D (5000 grams per 9000 meters of PET fiber bundles under conventional moisture regain) and 300 PET fibers in a bundle with a line density of 3000 D (3000 grams per 9000 meters of PET fiber bundles under conventional moisture regain) as materials and weaved them using two different knitting methods.

The full-set threading warp knitting method (A) can produce braided fabrics with a smoother surface and dimensional stability. For this knitting method, we used an independent improved Raschel II two-bar full-width weft insertion warp knitting machine (Haining Wellington New Material Co., Zhejiang, China). The width was reduced to 120** **cm, and the horizontal density (*P*_*a*_) and longitudinal density (*P*_*b*_) were set at 4/cm and 8/cm, respectively. The front bar used polyester fiber with 10% elastic module and 2.0** **cN/dtex breaking strength. The back bar used two high-purity (90% crystalline) PET fibers of different linear densities (3000 D, 5000 D). The machine speed was 3000** **r/min. The 5000 D and 3000 D PET-ALs, weaved using the full-set threading warp knitting method, were denoted by 5A and 3A, respectively.

The part-set threading warp knitting method (B) produces a braided fabric surface with relief and provides more sprang for improved porosity. For this knitting method, we used an RSJ4 multibar lace warp knitting machine (Haining Wellington New Material Co., Zhejiang, China). The width was 340** **cm, and the horizontal density (*P*_*a*_) and longitudinal density (*P*_*b*_) were set at 4/cm and 8/cm, respectively. The front bar used full-set threading with polyester fiber, and the back bar used part-set threading (50%) with high-purity (90% crystalline) PET fibers of different linear densities (3000 D, 5000 D). The 5000 D and 3000 D PET-ALs, weaved using the part-set threading warp knitting methods, were denoted by 5B and 3B, respectively.

The knitted PET-ALs were washed with a chemical cleaning agent, double distilled water, acetone, and absolute ethanol in an ultrasonic cleaner for 30** **min each to remove oil, dirt, and chemical residue.

**Figure 1:**
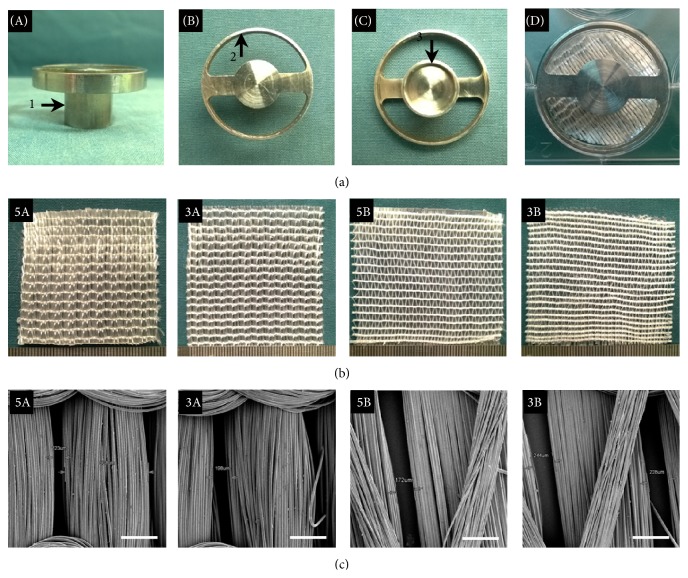
Gross view of the cell migration model (a-A, a-B, and a-C) and cell migration model with sheets (a-D). The gross view and SEM images of surface morphology of four kinds of PET-ALs (b and c). 5A: the linear density is 5000 D and the knitting method is full-set threading warp knitting; “5000 D” indicates 5000 grams weight of the 9000 meters PET fiber bundles under convention moisture regain; 3A: the linear density is 3000 D and the knitting method is full-set threading warp knitting; 5B: the linear density is 5000 D and the knitting method is part-set threading warp knitting; 3B: the linear density is 3000 D and the knitting method is part-set threading warp knitting; 1 indicates central cylinder (side); 2 indicates annular ring; 3 indicates central cylinder (underside). Scale bars: 500 *μ*m.

The cell migration model was fabricated using 316L medical stainless steel (Figures 1(a)-A, -B, and -C) with a height of 45** **mm. The diameter of the central cylinder was 10** **mm, and the annular ring at the top of the model was 35** **mm. The central cylinder and the annular ring were connected using an 8-mm-wide arm through which BMSCs could be seeded.

### 2.2. Graft Characterization

#### 2.2.1. Scanning Electron Microscopy

The morphologies and the pore diameters of the four types of PET-ALs were evaluated using an SEM (HITACHI-S4800 Scanning Electron Microscope, Japan) at a voltage of 5** **kV.

#### 2.2.2. Porosity

The open porosity of the four types of PET-ALs was evaluated based on the principle of liquid displacement (*n* = 6). The following equation was used to calculate the porosity of the PET-ALs:

(1) $$\begin{array}{c} \varphi =\frac{\left[\left(P_{T}-P_{S}\right)-\left(P_{R}-P_{P}\right)\right]/\rho_{{\text{H}}_{2}\text{O}}}{V_{N}-\left(P_{R}-P_{P}\right)/\rho_{{\text{H}}_{2}\text{O}}}\times 100\%, \end{array}$$

where *P*_*T*_ is the weight of the pycnometer when completely filled with distilled water and the tested PET-ALs, *P*_*S*_ is the weight of the dried pycnometer and the tested PET-ALs, *P*_*R*_ is the weight of the pycnometer and the residual water after the removal of the tested PET-ALs, *P*_*P*_ is the weight of the dried pycnometer, *V*_*N*_ is the nominal volume of the pycnometer (*V*_*N*_ = 50 mL), and *ρ*_H_2_O_ is the density of distilled water at the measurement temperature.

#### 2.2.3. Mechanical Properties

The four types of PET-ALs (*n* = 6) were rolled up into tube-like mesh structures to the specifications of an adult ACL. The combined length of the two free ends was 70.0 ± 0.2** **mm, and the diameter of the ligament section was 7.0 ± 0.5** **mm. The PET-ALs were tested using an Instron testing system (Model 4442, Instron Inc., MA). During the test, both ends of the PET-ALs were fixed with homemade clamps, and the distance between the clamps was 50.0 ± 0.2** **mm. The pretension was 2 N, and the elongation rate was 10** **mm/min. The stiffness and ultimate failure load were measured.

### 2.3. *In Vitro* Tests

#### 2.3.1. Isolation and Culture of BMSCs

Animal experiments were performed after approval by the Institutional Animal Care and Use Committee of The Fourth Military Medical University. Four-week-old Sprague-Dawley (SD) rats (male) were sacrificed by cervical dislocation. The rats were sterilized with 75% alcohol for 5** **min. The bilateral femur and tibia marrow cavities were exposed under aseptic conditions and washed with 10 mL of DMEM medium (Corning, USA) containing 10% fetal bovine serum (FBS, Gibco, USA), 100 U/mL penicillin, and 100 U/mL streptomycin (Sigma). The cell suspension was collected and centrifuged at 375 ×g for 5** **min. The supernatants were removed, and 5 mL of new DMEM medium was used to resuspend the sediment for inoculation into 25-cm^2^ plastic culture flasks. The medium was first changed at 24** **h and then changed every 3 d. The cell confluence was 70–80% after 7-8 d; the cells were subcultured, and the first parental generation (*P*_1_) was marked. Then, the BMSCs were subcultured every 3–5 d, and the numbering of the parental generations of BMSCs increased as the subculturing proceeded.

#### 2.3.2. Seeding of BMSCs

Before BMSC seeding, the four types of PET-ALs were sterilized with cobalt-60. The sheets were placed in an untreated 6-well plate and washed in PBS (phosphate buffered saline, pH 7.2–7.6) three times and in DMEM another three times. BMSCs from the third to fifth parental generation (*P*_3_–*P*_5_) were used for seeding. The adherent BMSCs were digested using 0.25% trypsin with 0.02% EDTA. The cell suspension was collected and centrifuged at 375 ×g for 5 min. The sediment was resuspended and seeded onto the sheets at 2 × 10^5^/mL in 6-well plate and cultured at 37°C in a humidified atmosphere containing 5% CO_2_.

#### 2.3.3. Evaluation of BMSC Adhesion and Morphology

The sheets (*n* = 3) were gently washed in PBS three times and fixed overnight in 2.5% glutaraldehyde solution after the cells had been seeded and cultured for 3 days. Then, the sheets were washed in DDW (doubly distilled water) 3 times and then dehydrated and coated with conductive coating. The morphologies of the BMSCs on the four types of PET-ALs were investigated via scanning electron microscopy (SEM, HITACHI-S4800, Japan), and the diameters of the four types of PET-ALs were measured.

For the BMSC adhesion studies, the F-actin cytoskeletons of the cells were stained with TRITC-labeled phalloidin (Cytoskeleton, USA), and the nuclei of the cells were stained with 4′,6-diamidino-2-phenylindole (DAPI, Sigma–Aldrich, St. Louis, MO, USA). After 3 days of incubation, the samples were fixed with 4% paraformaldehyde for 30 min, washed 3 times with PBS, permeabilized in 0.1% Triton X-100 for 10 min, and washed 3 times with PBS. After blocking nonspecific antibody binding with 1% bovine serum albumin (BSA) for 30 min at room temperature, the cells were stained with TRITC-labeled phalloidin (1 : 60 dilution in blocking solution) for 20 min and counterstained with DAPI for 10 min. The samples were mounted on coverslips and examined using a confocal laser scanning microscope (TCS SP5, Leica, Solms, Germany), and the areas of cells were measured using Image Pro Plus 6.0 (Media Cybernetics, Silver Spring, USA). Six different substrate fields were measured per sample, and three separate samples were measured for each type of implant.

#### 2.3.4. Cell Migration Analysis

The cell migration model was placed in a 6-well plate with PET-ALs, and then 2 mL of 5 × 10^5^/mL *P*_3_–*P*_5_ BMSCs was seeded on the plate. The model was removed after culturing for 24 h. Then, fluorescein diacetate (FDA) was added to the final concentrations of 100 *μ*g/mL. FDA can cross the membranes of living cells and appear green under fluorescence microscopy. The density of live cells was counted by Image Pro Plus 17.0 to confirm that the actual cell seeding densities were the same on different groups of ALs. After the cell migration model was removed and the cells were cultured for an additional 24 h, 48 h, and 72 h, the cells on the PET-AL sheets were fixed with Carnoy stationary liquid (methanol : glacial acetic acid = 3 : 1), followed by staining for 20 min with a working solution of Giemsa stain (Cat. number 32884, Sigma). The migration area of the BMSCs was established and computed using Image Pro Plus 6.0.

#### 2.3.5. Western Blotting

After the cell migration model was removed and the cells were incubated for 72 h, the BMSCs from the four types of PET-AL sheets were harvested using pancreatic enzyme and rinsed three times with PBS. RIPA buffer was added to lyse the cells. The lysates were centrifuged at 10^4^ ×g for 10 min at 4°C, and the protein in the supernatant was extracted. Equal amounts of protein (30 *μ*g) were separated by 10% SDS-PAGE and transferred to a PVDF membrane (poly-vinylidene fluoride, Bio-Rad). After blocking with 5% nonfat dry milk in Tris-buffered saline with Tween for 1 h, the membranes were incubated with the following antibodies overnight at 4°C: anti-RhoA (Santa Cruz Biotechnology, Inc., USA), anti-ROCK1 (Santa Cruz Biotechnology, Inc., USA), anti-pMLC (Cell Signaling Technology, Inc., USA), and anti-GAPDH (Santa Cruz Biotechnology, Inc., USA), followed by incubation with horseradish peroxidase-conjugated antibody for 1 h at room temperature. Signals were detected using the Western-Light Chemiluminescent Detection System (Peiqing, China). The integrated density values were calculated using Image Pro Plus 6.0.

### 2.4. *In Vivo* Tests

#### 2.4.1. Implantation Surgery Procedure

The animal experiments were performed strictly in accordance with the animal protocol approved by the Institutional Animal Care and Use Committee of The Fourth Military Medical University. Eighteen adult New Zealand rabbits (males, 12 weeks old, and 3.0 ± 0.4 kg) were subjected to extra-articular graft-to-bone healing surgery. The four types of PET-ALs were randomly implanted into two tunnels (*R* = 2 mm) in the distal femur and two tunnels (*R* = 2 mm) in the proximal tibia. Gentamicin (5 mg/kg) and penicillin (50 KU/kg) were injected for 3 consecutive days postoperatively. The rabbits were randomly sacrificed at 4 and 12 weeks after surgery.

#### 2.4.2. Mechanical Tests

Six specimens from each group were used for mechanical tests. Each graft was sutured using a No. 5 ETHIBOND suture and mounted onto a special jig to allow the graft to extend out of the tunnel. The test was performed using a Material Testing System Model 858 (MTS Systems, Minneapolis, MN) at an elongation rate of 2 mm/min. The ultimate failure load (N) and stiffness (N/mm) were measured.

#### 2.4.3. Histological Observations

All specimens were fixed in 80% ethanol for two weeks. Specimens were dehydrated in a graded ethanol series (70–100%) and then embedded in a methylmethacrylate (MMA) solution for 3 weeks. After polymerization of the MMA at 50°C for 24 h, pathological sections to the longitudinal axis and the horizontal axis were prepared using a band saw (Leica Microtome, Wetzlar, Germany), stained with Van Gieson, and observed under a light microscope (Leica LA Microsystems, Bensheim, Germany). Bone formation qualitatively analyzed the VG staining of pathological sections to the horizontal axis using Image Pro Plus 6.0, and the percentage of new bone formation was calculated using the following formula:

(2) $$\begin{array}{c} \frac{S_{a}}{\pi R^{2}-S_{b}}\times 100\%, \end{array}$$

where *S*_*a*_ is the area of the newly formed bone, *S*_*b*_ is the area of the graft, and *R* is the radius of the tunnel. The bonding of the graft to the adjacent tissue was analyzed by observing the VG staining of pathological sections to the longitudinal axis. Six different sections were measured per sample, and three separate samples were measured for each type of implant at each time point.

### 2.5. Statistical Analysis

The results were analyzed using GraphPad Prism 5 and are presented as the means ± SD for each group. At least three separate experiments were performed to obtain each set of results. *p* < 0.05 was considered significant in the paired Student's *t*-test.

## 3. Results

### 3.1. Characterization of Different PET-ALs

The characteristics of the four types of PET-ALs are summarized in Table 1. The pore diameter and the porosity of the 3000 D PET-ALs were significantly bigger than those of the 5000 D PET-ALs produced using the same knitting method (*p* < 0.05). Pore diameter and porosity were significantly higher in 3B than in the other three PET-ALs (*p* < 0.05). The pore diameters and porosities of 3A and 5B were not significantly different. The ultimate failure load and stiffness of 5A were significantly higher than those of the other three PET-ALs (*p* < 0.05), but there was no significant difference between 3A and 5B despite their different line densities (*p* > 0.05). SEM images of the four types of PET-ALs (Figure 1(b)) revealed that the two knitting methods produced different surface morphologies. 5A and 3A had a more smooth and cramped construction. These differences could also be observed in the gross photo (Figure 1(b)) of the four types of grafts.

### 3.2. Effect of Different PET-ALs on BMSC Adhesion and Morphology

**Figure 2:**
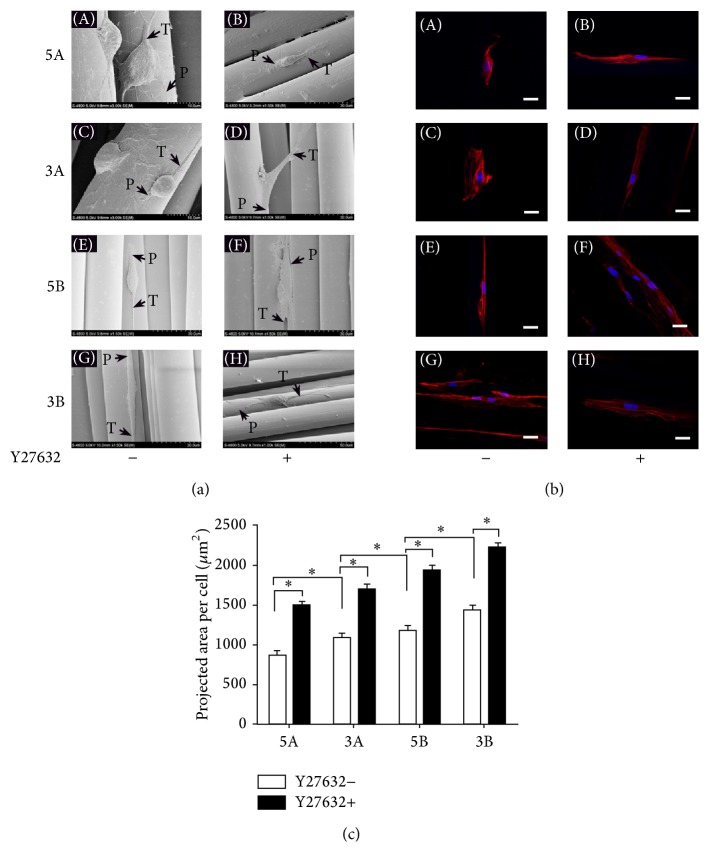
SEM images of BMSCs morphology in different groups after incubation for 3 days (a). Confocal laser scanning microscopy images (b) of BMSCs F-actin cytoskeletal (red, Rhodamine-phalloidin) morphology and nucleus (blue, DAPI) in different groups after 3 days of incubation. (c) Histogram of the cell projected cell area which was analyzed from the fluorescent images. P indicates cellular pseudopod, T indicates cellular tail, and ∗ indicates *p* < 0.05. Scale bars: 200 *μ*m.

The morphology of the BMSCs on the PET-ALs was observed after culturing for 72 h.  Figure 2(a) shows SEM images of BMSCs on the scaffolds. In those images, the BMSCs on 5A and 3A (Figures 2(a)-A and 2(a)-C) exhibited a more rounded appearance than those on 5B and 3B (Figures 2(a)-E and 2(a)-G). Figures 2(b)-A, 2(b)-C, 2(b)-E, and 2(b)-G present the immunostaining of the BMSCs on the scaffolds. Nucleus and F-actin were stained blue and red, respectively. As shown in Figure 2(c), the projected cell area of the BMSCs was significantly smaller on the 5A PET-ALs than on the other three PET-AL types (884.6 ± 156.8 *μ*m^2^ versus 1094.0 ± 116.9 *μ*m^2^, 1176.1 ± 176.5 *μ*m^2^, and 1450.3 ± 133.9 *μ*m^2^; *p* < 0.05).

### 3.3. Effect of Different PET-ALs on Cell Migration Area

**Figure 3:**
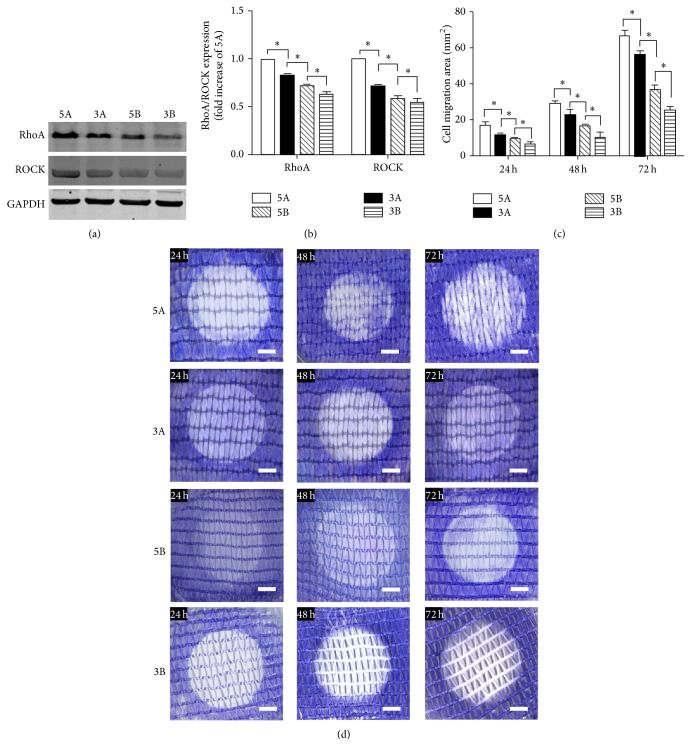
Western blot (a) and semiquantitative analysis (b) of RhoA and ROCK expression in BMSCs on different groups after incubation for 3 days. Stereomicroscope images (d) of Giemsa staining of different groups and histogram of cell migration area (c) which was analyzed from the stereomicroscope images. ∗ indicates *p* < 0.05. Scale bars: 2 mm (white).

After the cell migration model was removed and FDA staining indicated that there were no significant differences among the actual cell seeding densities on different group of ALs (live cell density: 908 ± 42/mm^2^), culturing was performed for 24 h, 48 h and 72 h, cell migration was evaluated by Giemsa staining (Figure 3(d)), and the cell migration area was measured (Figure 3(c)). At the 24-h time point, the migration area of the 5A PET-ALs (16.9 ± 3.4 mm^2^) was significantly larger than that of the other PET-AL types (*p* < 0.05), followed by 3A (11.8 ± 1.3 mm^2^, *p* < 0.05), 5B (9.5 ± 1.7 mm^2^, *p* < 0.05), and 3B (6.7 ± 1.6 mm^2^, *p* < 0.05). The migration area of 3A was significantly greater than that of 5B (*p* < 0.05). The migration area of 5B was significantly greater than that of 3B (*p* < 0.05).

### 3.4. Effect of Different PET-ALs on the Expression of RhoA/ROCK Pathway Components

Western blotting analysis was performed after the cell migration model was removed and culturing was performed for 72 h (Figures 3(a) and 3(b)). The expression levels of RhoA and ROCK in the BMSCs on the 5A PET-AL were set as the reference values and were significantly higher for 5A than for the other three PET-AL types (*p* < 0.05). The RhoA and ROCK expression levels were 1 on the 5A PET-ALs, and lower expression levels were observed on 3A (0.831 ± 0.024, 0.718 ± 0.032, *p* < 0.05), 5B (0.729 ± 0.017, 0.590 ± 0.045, *p* < 0.05), and 3B (0.636 ± 0.037, 0.555 ± 0.043, *p* < 0.05).

### 3.5. Effect of Y27632 on Cellular Migration

**Figure 4:**
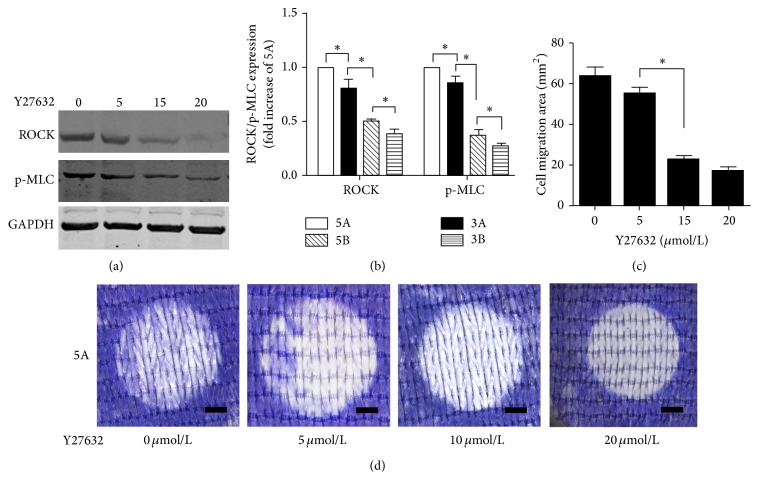
Western blot (a) and semiquantitative analysis (b) of ROCK and p-MLC expression in BMSCs on 5A PET-ALs after incubation for 72 h in the concentration gradient of 0, 5, 10, and 20 *μ*mol/L of Y27632. Stereomicroscope images (d) of Giemsa staining of 5A PE-ALs treated with different concentrations of Y27632 and histogram of cell migration area (c) which was analyzed from the stereomicroscope images. ∗ indicates *p* < 0.05. Scale bars: 2 mm.

We treated the 5A PET-ALs with Y27632 at concentrations of 0, 5, 10, and 20 *μ*mol/L for 72 h. Western blotting analysis (Figures 4(a) and 4(b)) revealed that the expression of ROCK and MLCP on the 5A PET-ALs decreased with increasing concentration of the inhibitor Y27632. The expression of ROCK and MLCP decreased significantly at Y27632 concentrations of 10 *μ*mol/L and 20 *μ*mol/L (*p* < 0.05), but they did not significantly decrease when the concentration of Y27632 was 5 *μ*mol/L (1 versus 0.804 ± 0.155 and 0.851 ± 0.114, *p* > 0.05), and the ROCK and MLCP expression levels were not significantly different between the 10 *μ*mol/L and 20 *μ*mol/L groups (0.505 ± 0.031 versus 0.393 ± 0.059 and 0.37 ± 0.087 versus 0.283 ± 0.028, *p* > 0.05). Cell migration was visualized by Giemsa staining (Figure 4(d)), and the cell migration area was measured (Figure 4(c)). The cell migration area of the 5A PET-ALs decreased significantly when the concentration of Y27632 was 10 *μ*mol/L or 20 *μ*mol/L (*p* < 0.05), but when the concentration of Y27632 was 5 *μ*mol/L (63.4 ± 7.7 mm^2^ versus 54.9 ± 5.6 mm^2^, *p* = 0.133), the cell migration areas did not significantly differ between the 10 *μ*mol/L and 20 *μ*mol/L groups (22.7 ± 2.8 mm^2^ versus 17.4 ± 2.7 mm^2^, *p* = 0.09). These results indicate that the appropriate concentration of Y27632 to inhibit cellular migration is 10 *μ*mol/L to 20 *μ*mol/L. We selected 15 *μ*mol/L as the concentration of Y27632 to treat BMSCs to observe the morphology of BMSCs on different PET-ALs. After treatment with 15 *μ*mol/L Y27632 for 3 days, the BMSCs displayed more filopodial extensions and more podosomes (Figures 2(a)-B, 2(a)-D, 2(a)-F, and 2(a)-H), and the projected cell areas (Figure 2(c)) of the four groups were significantly augmented (*p* < 0.05).

### 3.6. Effect of Different PET-ALs on Biomechanical Properties

**Figure 5:**
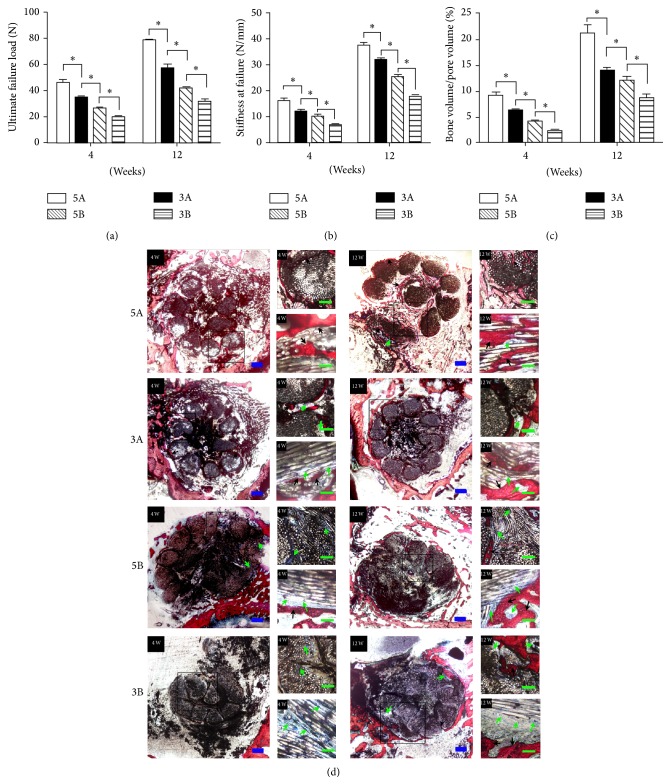
(a) The ultimate failure load of each group at 4- and 12-week time points. (b) The stiffness of each group at 4- and 12-week time points. (c) Histomorphometric analysis of new bone formation within each kind of implant. (d) The light microscope images of pathological sections of each kind of group. High-resolution microscope VG staining images of pathological sections to the longitudinal axis showed the details of graft-to-bone interface. The newly formed bone was stained red and fibrous tissue was stained dark blue. The black arrows indicate newly formed bone and the green arrows indicate fibrous tissue. ∗ indicates *p* < 0.05. Scale bars: 500 *μ*m (blue) and 200 *μ*m (green).

As shown in Figures 5(a) and 5(b), the 5A PET-AL exhibited an ultimate failure load of 46.42 ± 4.25 N and stiffness of 16.32 ± 2.52 N/mm at 4 weeks after surgery. These values were significantly higher than those of the other three groups (*p* < 0.05) and were followed by the 3A group (35.02 ± 2.09 N, 12.33 ± 1.13 N/mm), the 5B group (26.60 ± 2.53 N, 10.43 ± 1.44 N/mm), and the 3B group (20.15 ± 1.81 N, 7.2 ± 0.68 N/mm). At week 12 after surgery, the ultimate failure loads and stiffnesses of the four groups were significantly increased (*p* < 0.05). The biomechanical properties of the 5A PET-AL were still significantly higher than those of the other PET-ALs (78.25 ± 2.52 N, 37.72 ± 2.30 N/mm, *p* < 0.05), followed by 3A (57.48 ± 5.66 N, 31.98 ± 1.71 N/mm, *p* < 0.05), 5B (41.95 ± 2.42 N, 25.47 ± 1.77 N/mm, *p* < 0.05), and 3B (32.33 ± 2.59 N, 18.15 ± 1.17 N/mm).

### 3.7. Effect of Different PET-ALs on Bone Regeneration and Osseointegration

Von-Gieson (VG) staining was used to assess the implant-to-bone healing of the four types of PET-ALs at 4 and 12 weeks (Figure 5(d)). After 4 weeks, we observed that more regenerative bone (red) had integrated into the implant in the 5A PET-AL group (9.21 ± 1.35%), but little newly formed bone was integrated in the 3A PET-AL group (6.37 ± 0.53%). The least regenerative bone was observed in the 5B and 3B PET-AL groups (4.15 ± 0.54%, 2.41 ± 0.46%). High-resolution microscope images of VG staining of pathological sections to the longitudinal axis of the 5A PET-AL revealed that the regenerative bone bonded tightly to the 5APET-ALs, and no inflammatory reaction was observed. In the images of the VG staining of pathological sections to the horizontal axis of 5A PET-AL, we observed more regenerative bone and less fibrous tissue (dark blue) in the inner region of the PET-AL, whereas less regenerative bone and more fibrous tissue (dark blue) were observed in the inner region of the 3A, 5B, and 3B PET-ALs. After implantation for 12 weeks, the amount of regenerative bone was significantly higher in the 5A PET-AL group (21.32 ± 3.37%) than the other three groups (14.11 ± 1.15%, 12.1 ± 1.53%, 8.76 ± 1.48%, *p* < 0.05). High-resolution microscope images of VG staining of pathological sections to the longitudinal axis of the 5A PET-AL revealed that new bone was formed between the fibers of the 5A PET-ALs, while more fibrous tissue was observed in the other three PET-AL groups.

## 4. Discussion

Osseointegration into the insertion site is an important factor in artificial ligament use [35]. Many studies of materials and surface modification have sought to improve the osseointegration of artificial ligaments [36–38], but few have examined the effects of artificial ligaments with different porous structures on bone regeneration. Such studies are necessary to improve the biocompatibility and clinical performance of artificial ligaments.

In this study, four types of artificial ligaments prepared from nonbiodegradable and avirulent polyethylene terephthalate (PET) fibers were used to investigate the effects of porous structures on cellular migration. We observed that 5A PET-ALs were the most suitable for BMSC migration and bone regeneration; the other three groups exhibited smaller cellular migration area and more soft tissues (dark blue) than bone regeneration (Figure 5(d)). Scar tissue infiltration hinders the osseointegration of artificial ligaments [39, 40]. Since the regenerative bone bonded tightly to the four types of PET-ALs (Figure 5(d)), the biomechanical properties of the four PET-ALs groups were positively correlated with the quantity of the newly formed bone. Interestingly, we did not observe significant differences in pore diameter and porosity between 3A and 5B, but the migration area and bone regeneration of 3A were significantly higher than those of 5B. SEM images and gross photos (Figures 1(b) and 1(c)) revealed differences between the surface topographies of the A PET-ALs and the B PET-ALs; the A PET-ALs had a smoother surface, whereas the B PET-ALs had a more rugged surface. We demonstrated that a rugged surface has a main negative effect on cellular migration. A strong correlation among decreased pore diameter, decreased porosity, and increased cellular migration area was observed in the four PET-AL groups (Table 1). We demonstrated that PET-ALs with lower pore diameter and lower porosity had a larger cellular migration area (Figure 3(d)), and the inhibition of BMSC migration by the porous structure of the artificial ligaments increased as the porosity and pore diameter increased. In accordance with our findings, extensive research has demonstrated that increasing pore diameter has a negative effect on bone ingrowth [41–43]. Although some studies have suggested that increasing the porosity of scaffolds in combination with the pore diameter provides a larger surface area for the attachment and proliferation of cells [20], cells appeared less mobile with the combined effect of pore diameter increasing (Figure 3(d)). Although previous studies have demonstrated that cellular behavior is affected by the surface topography, pore diameter, and porosity of materials, the underlying biological mechanisms have remained elusive [44, 45].

The RhoA/ROCK signaling pathway is part of the Rho family, which is important for limiting membrane protrusions [46, 47]. This pathway retracts the rear of the cell by severing existing F-actin filaments and phosphorylating myosin light chain phosphatase (MLC) to regulate cell migration [48]. In the present study, western blotting analysis (Figures 3(a) and 3(b)) verified that the expression of RhoA and ROCK differed among the grafts. Expression was significantly higher in the 5A group than the other three groups, followed by the 3A group, 5B group, and 3B group. Researches had reported that ROCK negatively regulates membrane protrusion to promote migration [33]. Cells with high ROCK expression exhibit a rounded appearance, and the inhibition of ROCK augments the projected cell area. In our study, SEM images (Figure 2(a)) of BMSCs in the A groups exhibited a more rounded appearance than those of the B groups, and immunostaining images revealed that the projected cell area of BMSCs was significantly smaller in the A group than the B group. Our results indicate that differences in BMSC migration may be at least partly attributable to the expression of the RhoA/ROCK signaling pathway. This conclusion is further supported by the morphology, projected cell area, and migration area of BMSCs treated with Y27632, a specific inhibitor of ROCKs [34]. We observed increased lamellipodia and filopodia formation by BMSCs in the SEM images of the four types of PET-ALs; immunostaining also revealed increased F-actin staining after the BMSCs were treated with Y27632, and the projected cell areas were significantly increased in the four groups.

Based on the above findings, different weaving methods that lead to different surface topographies, diameters, and porosities of PET-ALs may regulate BMSC migration through the Rho/ROCK signaling pathway. Weaving PET-ALs using different methods is a potential strategy for improving the biocompatibility of PET-ALs. An ideal ACL prosthesis should be biocompatible and should mimic the mechanical properties of the native ACL [14, 15]. In our study, the 5A PET-AL was the most suitable for BMSC migration and bone regeneration. The ultimate failure load and the stiffness of native ACLs are 2160 ± 157 N and 242 ± 28 N/mm; thus, the 5A PET-AL has mechanical properties similar to those of the native ACL. We demonstrated that 5A is an ideal ACL prosthesis for ACL reconstruction.

Cellular migration can also be promoted by modification of artificial ligaments by growth factors [49] and bioactive proteins [50], which effectively improve bone tissue ingrowth to promote graft-to-bone healing [51, 52]. In future studies, we should use an improved knitting method in combination with surface modification to increase the biocompatibility of the PET-ALs.

## 5. Conclusions

In this study, four types of PET-ALs with different surface topographies, diameters, and porosities were investigated. The 5A PET-ALs were the most suitable for BMSC migration and effectively promoted graft-bone healing. Moreover, the beneficial effect of the 5A PET-AL on cellular migration was abolished by blocking the Rho/ROCK signaling pathway with Y27632. Our study explicitly demonstrates that the migration ability of BMSCs is regulated by differences in the porous structure of artificial ligaments via the Rho/ROCK signaling pathway and establishes a proper knitting method and line density of artificial ligament to attain improved biocompatibility and clinical performance.

## Figures and Tables

**Table 1:** Characterization of different PET-ALs (*n* = 6, mean ± SD).

Implant	Pore diameter (*μ*m)	Porosity (%)	Ultimate failure load (kN)	Stiffness (N/mm)
5A	153.4 ± 10.2	55.2 ± 0.9	6.6443 ± 0.1015	215.3 ± 15.6
3A	190.7 ± 12.1^∗^	72.4 ± 3.2^∗^	4.886 ± 0.0688^∗^	86.4 ± 3.2^∗^
5B	180.4 ± 15.9^∗^	68.3 ± 1.5^∗^	4.4210 ± 0.0980^∗^	92.6 ± 7.4^∗^
3B	245.3 ± 17.3^∗†#^	79.5 ± 2.2^∗†#^	2.5668 ± 0.0794^∗†#^	32.7 ± 2.7^∗†#^

^∗^Compared with 5A group: *p* < 0.05; ^†^Compared with 3A group: *p* < 0.05; ^#^Compared with 5B group: *p* < 0.05.

“5” indicates that the PET-ALs were weaved with 5000 D PET fiber bundles. “5000 D” indicates 5000 grams weight of the 9000 meters PET fiber bundles under convention moisture regain.

“3” indicates that the PET-ALs were weaved with 3000 D PET fiber bundles.

“A” indicates that the PET-ALs were weaved in full-set threading warp knitting method.

“B” indicates that the PET-ALs were weaved in part-set threading warp knitting method.
